# Supplementary material for: Decoupled few-femtosecond phase transitions in vanadium dioxide
Source: Nat Commun. 2025 Apr 19;16:3714. doi: 10.1038/s41467-025-58895-z (PMC12009403; doi:10.1038/s41467-025-58895-z)
Supplement: Supplementary file 1 — Supplementary Information [file 41467_2025_58895_MOESM1_ESM.pdf]

Supplementary Information for *Decoupled few-femtosecond phase transitions in vanadium dioxide*

Christian Brahms[1], Lin Zhang[2], Xiao Shen[3], Utso Bhattacharya[2,4], Maria Recasens[2], Johann Osmond[2], Tobias Grass[5,6], Ravindra W. Chhajlany[7], Kent A. Hallman[8], Richard F. Haglund[8], Sokrates T. Pantelides[8,9], Maciej Lewenstein[2,10], John C. Travers[1], and Allan S. Johnson[11†]*

[1]School of Engineering and Physical Sciences, Heriot-Watt University, Edinburgh EH14 4AS, UK
[2]ICFO - Institut de Ciencies Fotoniques, The Barcelona Institute of Science and Technology, Avenida Carl Friedrich Gauss 3, E-08860 Castelldefels (Barcelona), Spain
[3]Department of Physics and Materials Science, University of Memphis, Memphis, Tennessee 38152, USA
[4]Institute for Theoretical Physics, ETH Zurich, 8093 Zurich, Switzerland
[5]DIPC - Donostia International Physics Center, Paseo Manuel de Lardizabal 4, 20018 San Sebastian, Spain
[6]IKERBASQUE, Basque Foundation for Science, Plaza Euskadi 5, 48009 Bilbao, Spain
[7]Institute of Spintronics and Quantum Information, Faculty of Physics and Astronomy, Adam Mickiewicz University, 61-614 Poznań, Poland
[8]Department of Physics and Astronomy, Vanderbilt University, Nashville, Tennessee 37235, USA
[9]Department of Electrical and Computer Engineering, Vanderbilt University, Nashville, Tennessee 37235, USA
[10]ICREA, Passeig Lluis Companys 23, 08010 Barcelona, Spain
[11]IMDEA Nanoscience, Calle Faraday 9, 28049, Madrid, Spain
[*]c.brahms@hw.ac.uk
[†]allan.johnson@imdea.org

This document contains Supplementary Figures 1-10.

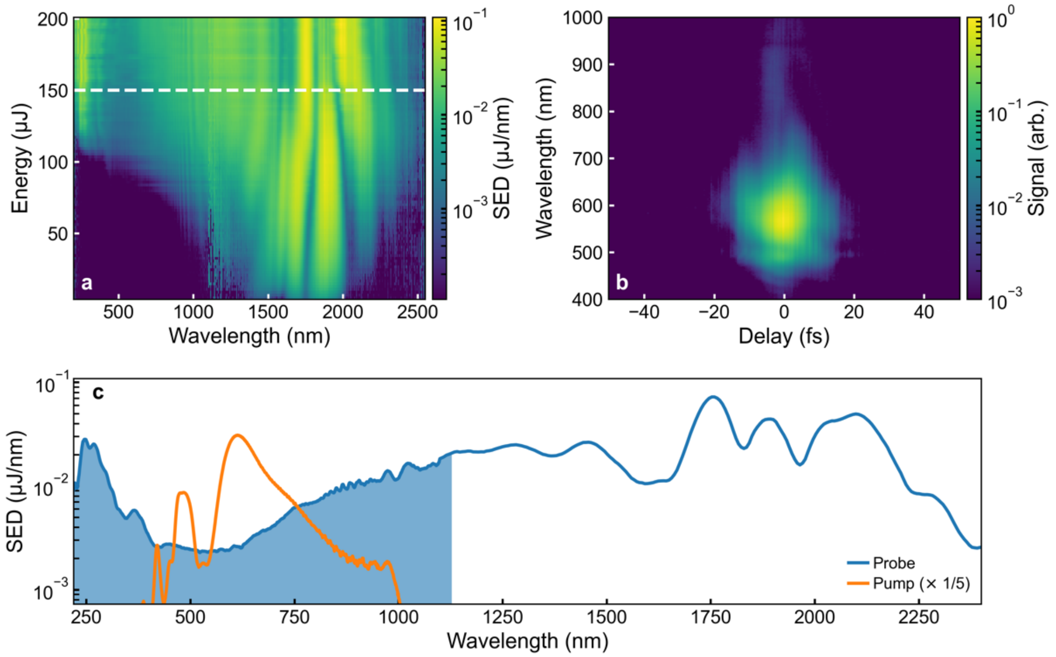

Supplementary Figure 1: Pump and characterisation. **a**, Energy-dependent output probe spectrum on a logarithmic colour scale. The white dashed line indicates the energy used in the experiments (150 μJ). **b**, In-situ self-diffraction XFROG trace of the pump pulse. **c**, Probe spectrum at 150 μJ (blue) and the spectrum of the pump pulse (orange). The shaded area indicates the spectral region covered in the pump-probe measurements in Fig. 2.

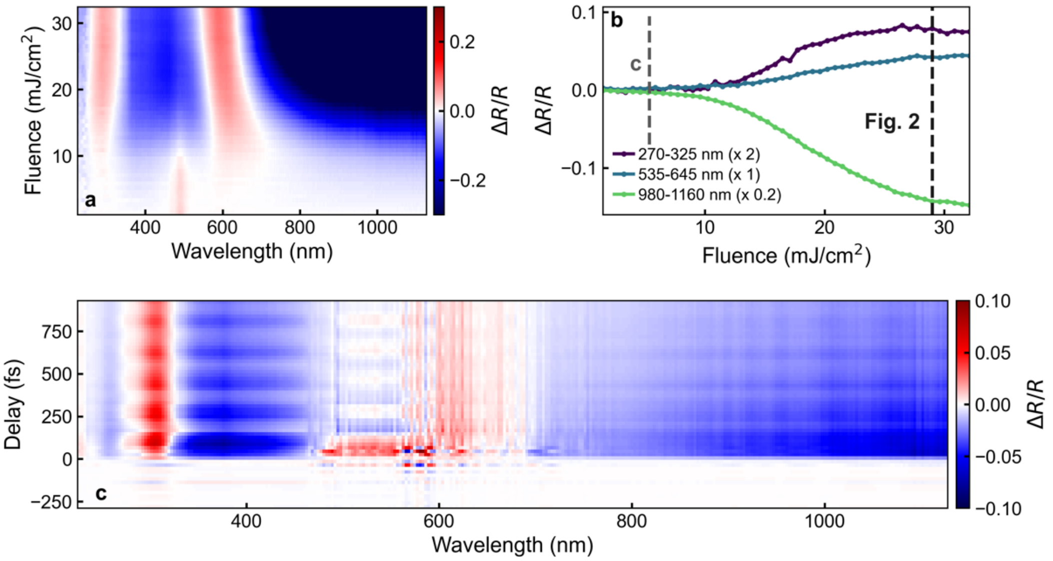

Supplementary Figure 2: Fluence threshold for the IMT. **a**, Fluence-dependent differential reflectivity at a fixed pump-probe delay of 1 ps. The incident fluence is determined as $F = 4E/(\pi w_x w_y)$, where E is the on-target pulse energy and w_x and w_y are the FWHM focal spot widths in the x and y axes, respectively. The positive feature around 490 nm at low fluence is an artefact due to pump scatter. **b**, Lineouts of the data shown in **a** in three different wavelength bands, clearly showing the onset of the IMT around 10 mJ/cm^2. The vertical dashed line indicates the fluence used for the pump-probe experiments shown in Fig. 2, which for the penetration length of the pump and our film thickness ensures excitation across the phase transition in the full depth of the VO_2. **c**, At fluences below 10 mJ/cm^2 we see clear 6 THz coherent phonons modulating the temporal response across the entire spectrum. As shown in Figure 2, no such signature is observed at high fluence, indicating a structural phase transition.

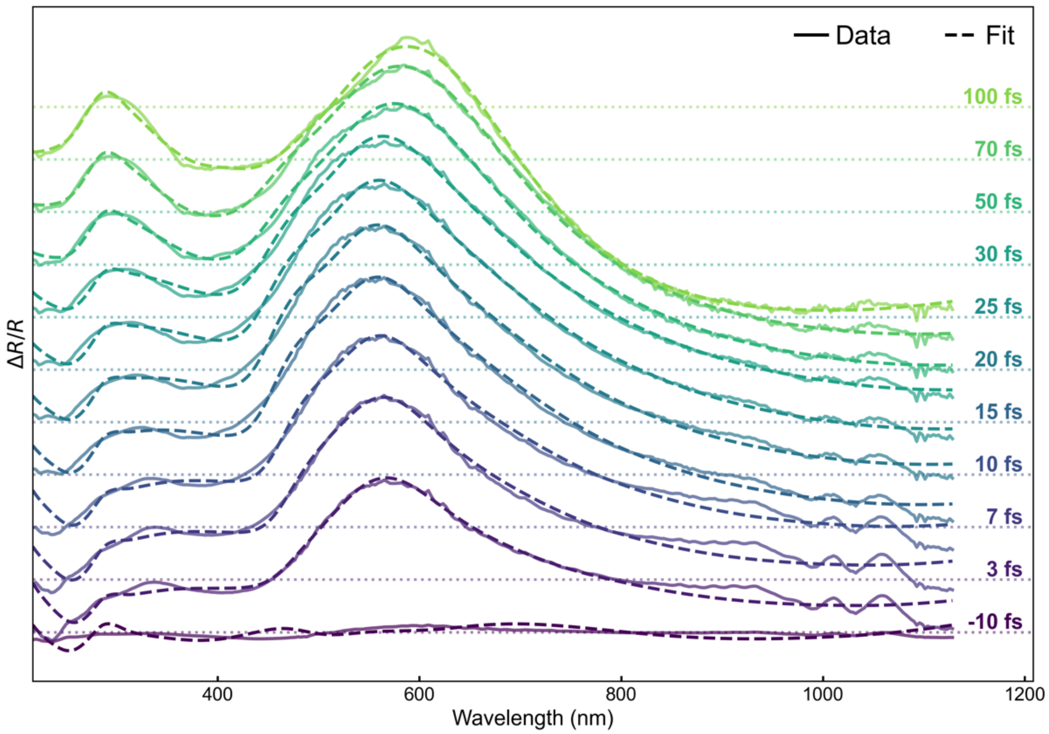

Supplementary Figure 3: Detailed comparison between fit and experiment. Solid lines show the experimental data from Fig. 2**a** at selected pump-probe delays as indicated on the right-hand side. Dashed lines show the fit from Fig. 2**c** at the corresponding delay.

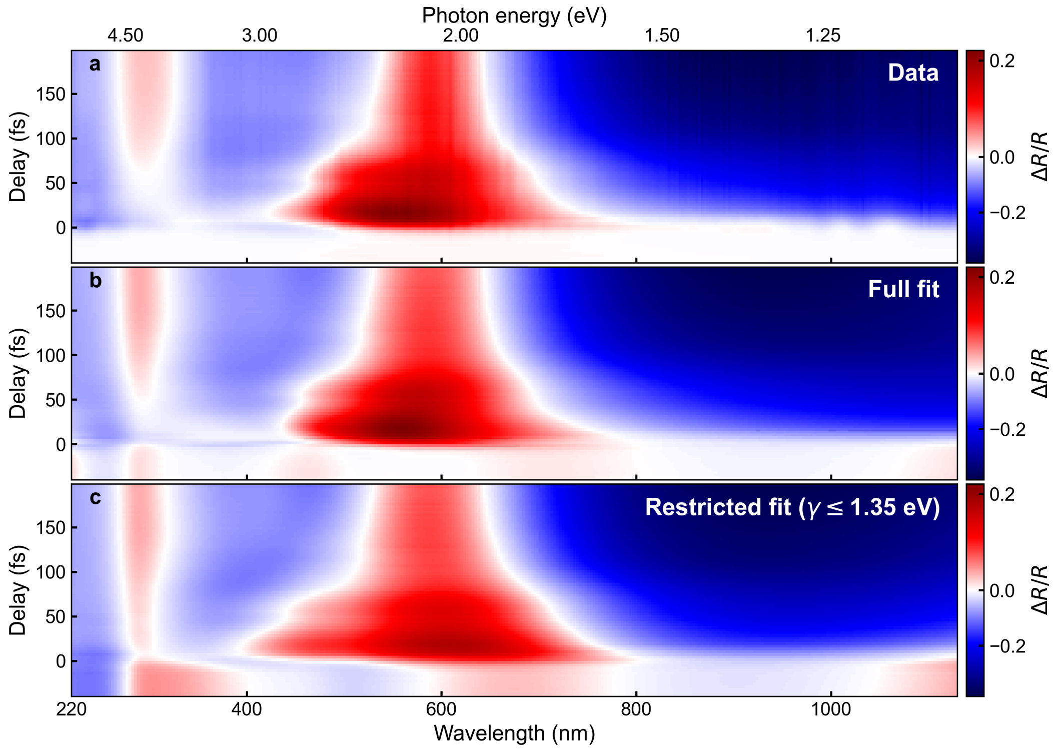

Supplementary Figure 4: Fit with clamped damping. a Experimental data (same as Fig. 2**a**). **b** Full fit with free parameters as discussed in the main text (same as Fig. 2**c**). **c** Result of fitting with the Drude damping term γ clamped to 1.35 eV (slightly above the value in the rutile phase through heating or at long time delays). The fit with restricted damping is clearly lacking, indicating that the response cannot be explained without the damping contribution.

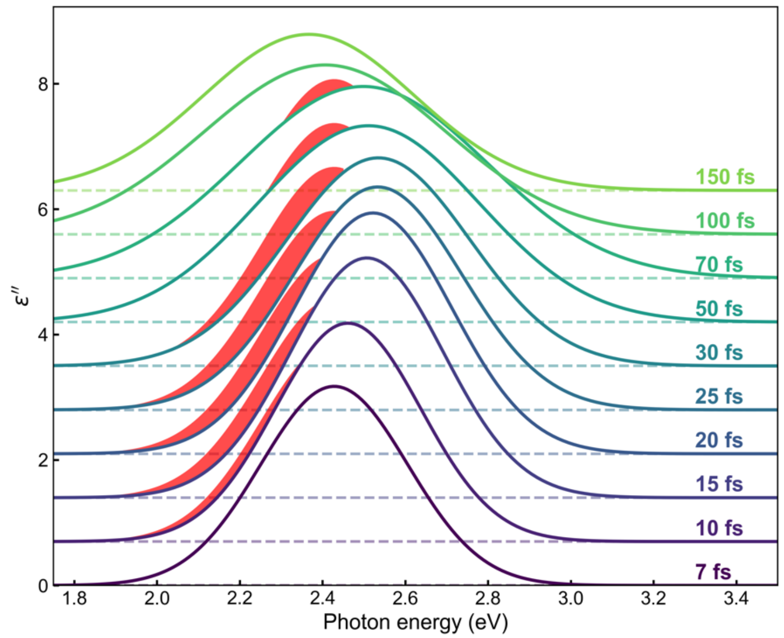

Supplementary Figure 5: Shift in the $d_\parallel$ transition and density-of-states bottleneck. Each line shows the imaginary part of the permittivity ε'' due to only the $\mathrm{O}_{2p} \rightarrow d_\parallel$ transition at one delay. Compared to the state immediately after photoexcitation, the transition shifts around 0.2 eV higher in energy and the transition strength on the low-energy side is reduced (red shaded areas). This is clear evidence of a transient reduction in the density of states just above the Fermi level.

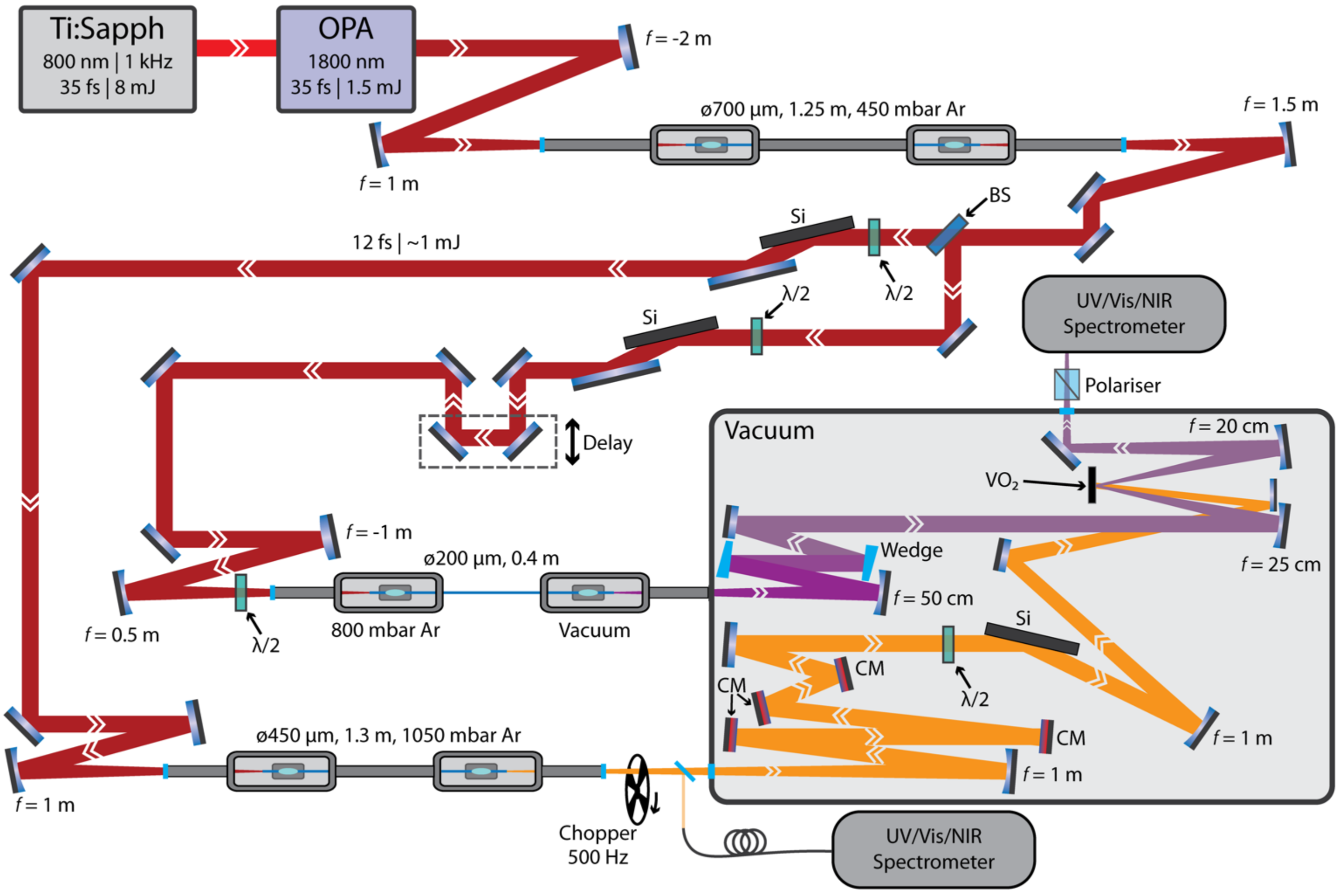

Supplementary Figure 6: Layout of the experimental apparatus. 35 fs laser pulses at 800 nm are converted to 1800 nm wavelength in an optical parametric amplifier (OPA) and compressed to 12 fs duration in a first gas-filled hollow capillary fibre (HCF). After beamsplitting and attenuation, pump and probe pulses are generated in separate second-stage HCFs. Inside a vacuum chamber, pump and probe pulses are overlapped in space in time on a VO_2 sample. The reflected probe is analysed by a spectrometer outside the chamber. A second synchronised spectrometer measures the pump spectrum before the vacuum chamber and after a chopper wheel to reference the pump-on/pump-probe shots.

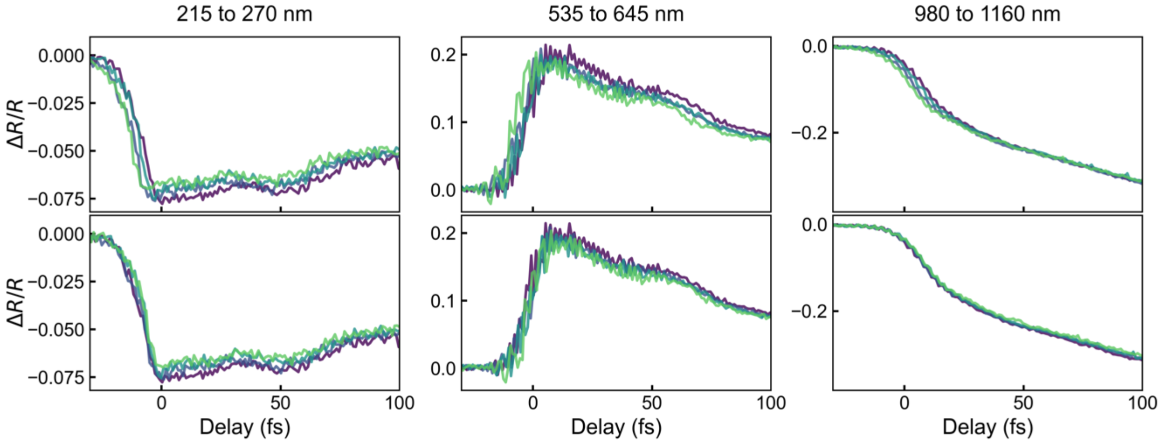

Supplementary Figure 7: Delay axis correction. Each column shows one of the line-outs shown in Fig. 2**b**. Each line corresponds to one of the four subsequent delay scans in the experiment. The top row shows line-outs from the raw data and the bottom row shows line-outs from the delay-corrected data.

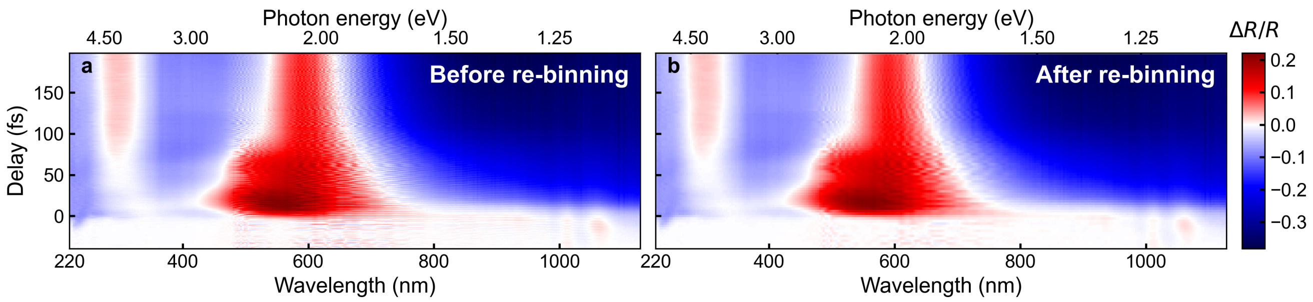

Supplementary Figure 8: Delay-axis rebinning. a, Delay-corrected and averaged scans with full delay resolution (1 fs steps). **b**, The data in **a** after re-binning along the delay axis by a factor 3 to remove fast coherent artefacts.

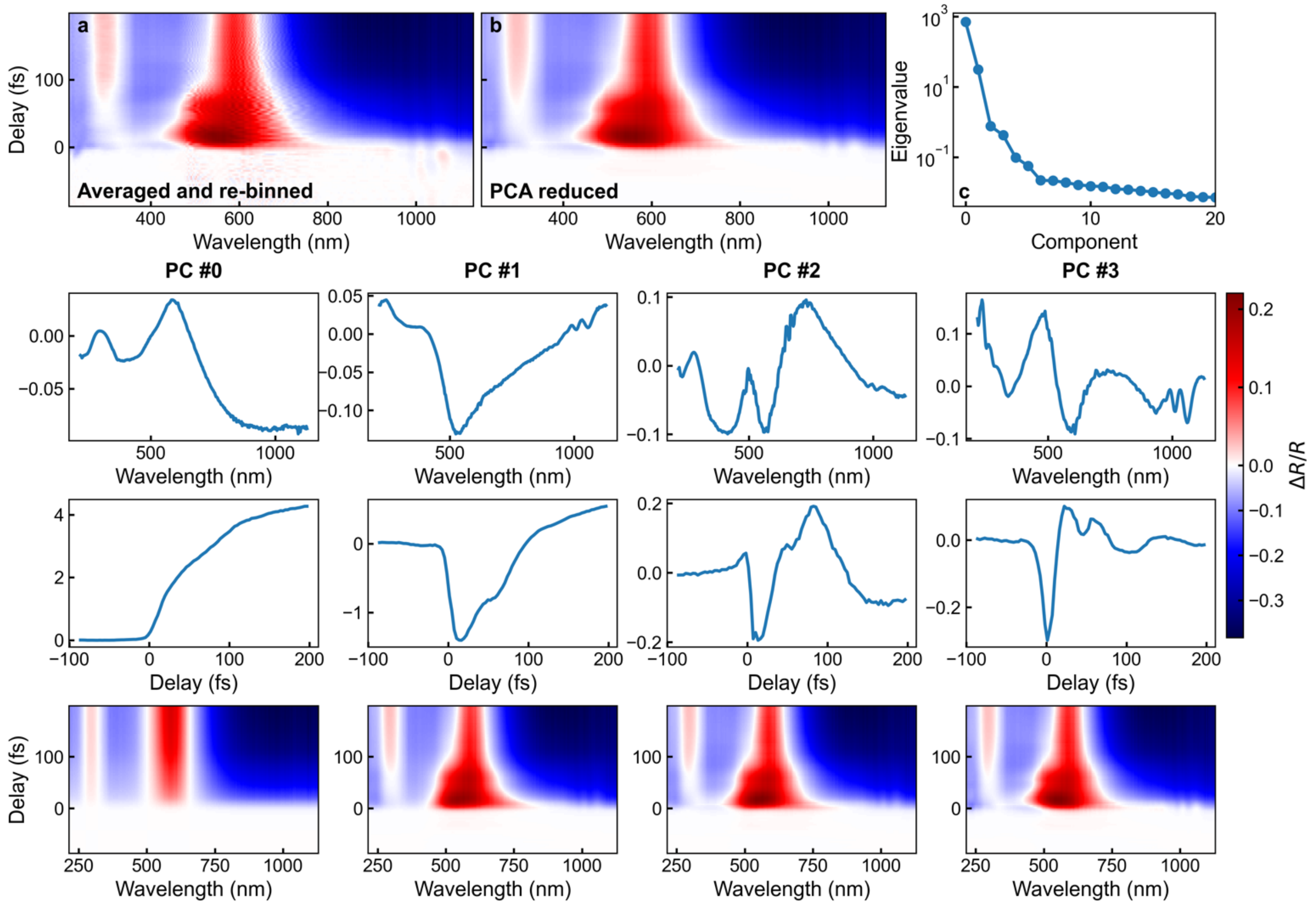

Supplementary Figure 9: Principal-component analysis. Top: differential reflectivity after re-binning (**a**) and after PCA reduction (**b**). Bottom: each column shows the wavelength-axis eigenvector (first row) and delay-axis eigenvector (second row) of one principal component. The bottom row shows the cumulative result of the PCA reconstruction, showing that with 4 components, all essential features of the data are reproduced. The colour bar applies to all false-colour plots.

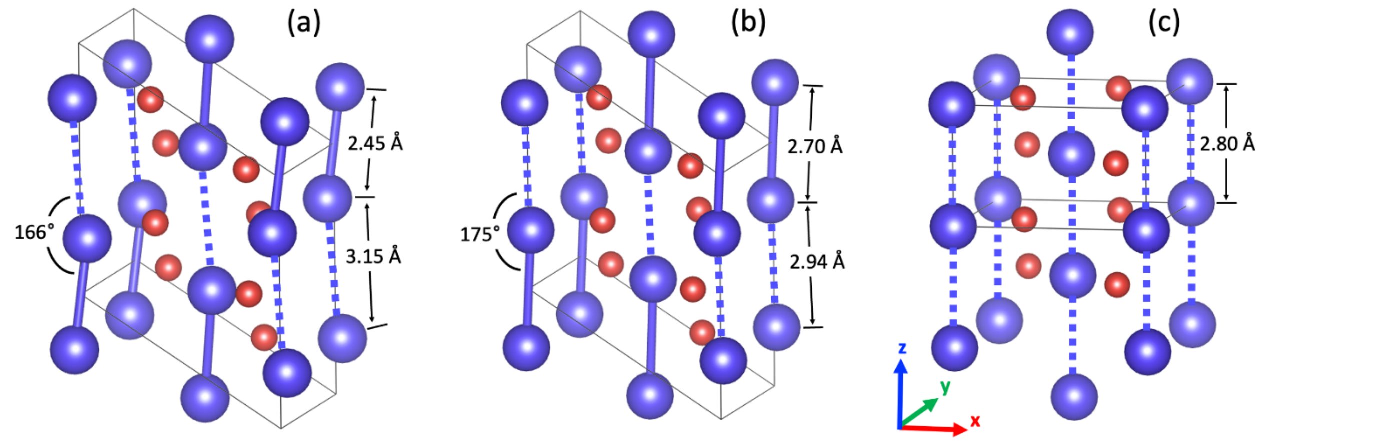

Supplementary Figure 10: The DFT calculated lattice structure of the M1 (a), M0 (b), and R (c) phases of VO_2 with vanadium ions in blue, oxygens in red and the vanadium-vanadium bonds along the rutile c-axis direction indicated with dashed (solid) lines for the long (short) bonds. Shown are the relevant long and short vanadium-vanadium bond lengths as well as angles. Converted from bond lengths and angles into the X1 and X2 nomenclature used in the main text (deviations from the R phase positions), we obtain phase X_1=0.187 Å and X_2=0.168 Å in the M1 phase, X_1=0.051 Å and X_2=0.061 Å in the M0 phase, and X_1= X_2=0 in the R phase.
